# CardioMEMS guided heart failure management in cardio-oncology patients: a tertiary care cancer center experience

**DOI:** 10.1186/s40959-025-00355-0

**Published:** 2025-07-18

**Authors:** Abdelrahman Ali, Maximillian Bourdillon, Hyeon-Ju Ryoo Ali, Juhee Song, Efstratios Koutroumpakis, Poonam Jewani, Shaden Khalaf, Ihab Hamzeh, Salil Kumar, Nicolas L. Palaskas, Jean-Bernard Durand, Cezar Iliescu

**Affiliations:** 1 Department of Cardiology, Division of Internal Medicine, The University of Texas MD Anderson Cancer Center, 1400 Pressler Street, Unit 1451, Houston, TX 77030 USA; 2 Division of Cardiology, Department of Internal Medicine, McGovern Medical School, Houston, TX USA; 3 Department of Biostatistics, The University of Texas MD Anderson Cancer Center, Houston, TX USA

**Keywords:** CardioMEMS, Pulmonary artery pressure sensing devices, Heart failure, Cardio-oncology

## Abstract

**Aims:**

Cancer patients and survivors are at increased risk of developing heart failure (HF) and heart failure hospitalization (HFH). Yet, the utilization of wireless pulmonary artery pressure sensing devices (PAPSD), like CardioMEMS, in this group is limited.

**Objectives:**

We aimed to explore the utilization of CardioMEMS in managing HF among oncology patients.

**Methods:**

We conducted a single-center retrospective study reviewing consecutive patients implanted with the CardioMEMS device between November 11, 2015, and February 21, 2023. We analyzed the device's impact on pulmonary artery pressures and HFH using statistical methods including Cox regression models and correlation studies between NT-proBNP levels and hemodynamic parameters.

**Results:**

The study included 28 patients, with hypertension (78%) and hyperlipidemia (78%) as prevalent comorbidities. Most patients had heart failure with preserved ejection fraction (64%). Post-implantation, we observed a reduction in HFH and improvements in pulmonary artery pressures. Cox regression identified prior HFH and elevated pulmonary artery systolic (PAS) and diastolic pressures (PAD) as risk factors for repeat HFH (HR: 1.24, 1.04, 1.07, respectively). Biomarker analysis showed a moderate positive correlation between NT-proBNP and PAD, indicating that higher levels are associated with increased hospital admissions. The device was safe with no sensor failures reported.

**Conclusions:**

CardioMEMS shows potential in improving HF management in cancer patients, reducing HFH and enhancing pulmonary artery pressure profiles. These preliminary results advocate for further, larger-scale prospective studies to confirm the benefits and integrate CardioMEMS into cardio-oncology care.

**Graphical Abstract:**

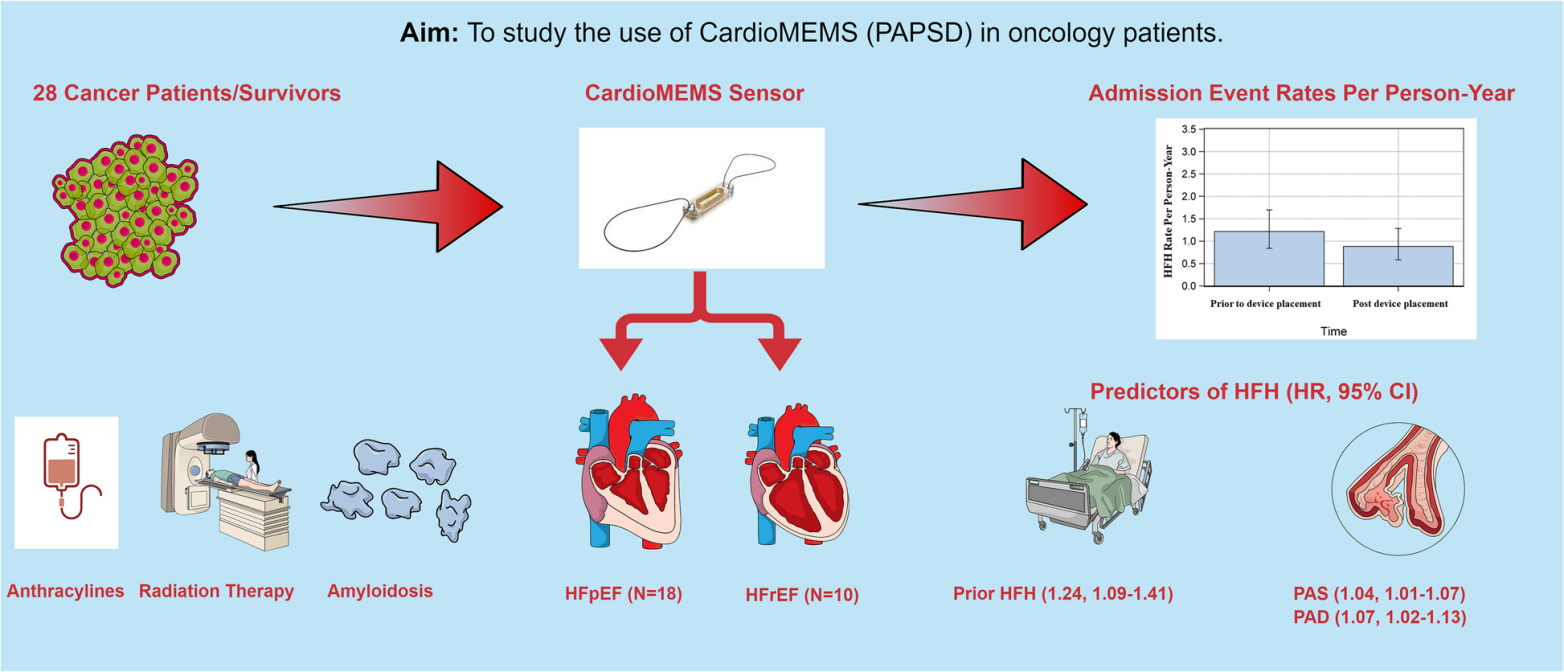

**Supplementary Information:**

The online version contains supplementary material available at 10.1186/s40959-025-00355-0.

## Introduction

Heart failure (HF) continues to be a major contributor to morbidity and mortality, resulting in almost 6.5 million hospital days annually in the United States [1]. Patients with active cancer and cancer survivors are at increased risk of developing HF, especially in conjunction with non-Hodgkin lymphoma, leukemia, multiple myeloma, and lung malignancies [2, 3]. As cancer is second only to cardiovascular disease as the leading cause of death worldwide, there is great unmet need in understanding the intersection between HF and cancer [4]. Patients with HF and cancer share many common risk factors associated with inflammation, such as hypertension, obesity, diabetes, dyslipidemia, and tobacco smoking [5, 6].

Due to complex pathology, increased frailty and reduced resilience to treatment challenges, patients with cancer are at an increased risk for heart failure hospitalizations (HFH) [7]. It is imperative to implement advanced care strategies to enhance their cancer treatment and mitigate the risk of HFH [8]. The Centers for Medicare & Medicaid Services standards target a reduction in 30-day readmission rates for heart failure to less than 20%, which is considered a gold standard for reimbursement [9]. Over the past decade, pulmonary artery pressure monitoring through wireless pulmonary artery pressure sensing devices (PAPSD), such as CardioMEMS, has demonstrated effectiveness in reducing HFH in patients with heart failure with reduced ejection fraction (HFrEF) and those with heart failure with preserved ejection fraction (HFpEF) [10–12], with real-world, post-marketing surveillance studies confirming this benefit [13, 14]. Furthermore, this technology not only reduces HFH rates but is associated with improved quality of life and may decrease mortality rates in patients with HFrEF [15, 16]. Cost-effectiveness analyses from Western Europe have substantiated the economic benefits of incorporating PAPSD in addition to the standard of care [17, 18]. However, patients concurrently undergoing cancer treatment or with cancer therapy related cardiac dysfunction (CTRCD) have been excluded from previous studies and reports [19–21].

To date, the use of CardioMEMS in patients with cancer has been limited to a handful of individual case reports [22, 23]. To address this knowledge gap, here we report our single-center experience with the use of CardioMEMS in managing HF in cancer patients or those experiencing CTRCD.

## Methods

### Study cohort

We conducted a single-center retrospective review of consecutive patients who underwent CardioMEMS implantation between November 11, 2015, and February 21, 2023 for clinical management of heart failure. CardioMEMS were implanted in the patient’s pulmonary artery in accordance with its commercial approval by the US Food and Drug Administration and previously published methods, with device calibration with hemodynamics obtained and right heart catheterization done at the time of implant [10]. All patients have received antiplatelet therapy comprising of aspirin for a minimum of one month following the placement of the device, unless patient was on systemic anticoagulation. All patients were treated with maximally tolerated doses of guideline-directed medical therapy, and diuretics were adjusted based on weekly remote reviews of CardioMEMS data. These reviews were conducted by trained advanced practice providers familiar with each patient’s clinical course, in collaboration with the supervising cardiologist. Titration decisions were individualized according to target PAD, renal function, age, and other comorbidities**.** Baseline demographics, clinical characteristics, echocardiographic parameters, cardiac biomarkers, invasive hemodynamics, and clinical events were extracted from the electronic medical record by individual chart review.

HFH was defined as any unplanned hospital admission lasting at least 24 h due to worsening heart failure, characterized by clinical symptoms (e.g., dyspnea, fatigue), objective signs (e.g., weight gain, peripheral edema, elevated jugular venous pressure), elevated cardiac biomarkers (e.g., NT-proBNP), and requiring intravenous diuretic therapy [19] Device-/system-related complication (DSRC) was defined by the presence of one of the following: catheter site hematoma, catheter site hemorrhage, device dislocation, device malfunction, arterial injury, and pseudoaneurysm. Event adjudication was performed by two separate individuals (A.A) and (M.B). Informed consent was waived given the retrospective design of the study.

### Statistical analysis

Patients’ demographic and baseline characteristics were summarized by descriptive statistics: mean (SD) or median (IQR) for continuous variables, and frequency (%) for categorical variables. Admission event rates per person-year (defined as the number of admissions divided by follow-up time in person-years) before and after device implantation were estimated along with 95% confidence intervals (CIs). Admission event rates per person-year along with 95% CIs were evaluated further according to subgroups defined by cardiac indication and heart failure type.

Univariable and multivariable Cox regression models with time-varying covariates by Prentice, Williams, and Peterson total time models (PWP models) with common effects were fitted to assess the significance of hemodynamic and echocardiographic parameters on the risk of future HFH [24]. The mean cumulative number of admission events was estimated according to the number of HFH before device implantation, pulmonary artery systolic pressure (PAS) (normal vs. abnormal), pulmonary artery diastolic pressure (PAD) (normal vs. abnormal) and mean pulmonary artery pressure (mPAP) (normal vs. abnormal) based on PWP models with a common effect. Profile plots of PAS, PAD, and mPAP over time were plotted for patients with HFH after device placement and those without HFH admissions after device placement.

Scatter plots were plotted using (1) the mean NT-proBNP level and the mean PAD for each patient and (2) all repeated measures of NT-proBNP and PAD. Pearson correlation coefficient between mean NT-proBNP and mean PAD was calculated. Spearman partial correlation accounting for subject effect was calculated. Elevated NT-proBNP levels, PAS, mPAP, and PAD pressure were defined as > 400 pg/mL [25], > 35 mmHg, > 20 mmHg, and > 18 mmHg, respectively. A p-value less than 0.05 indicated statistical significance. SAS 9.4 (SAS Institute, Cary, NC) was used for data analysis.

## Results

### Baseline characteristics

The study cohort included 28 patients, whose characteristics are described in Table 1. The majority of patients in our study had history of hypertension and/or hyperlipidemia, while approximately half of the cohort had a history of coronary artery disease and chronic kidney disease. HFpEF was the predominant subtype of HF. With regards to oncologic history, hematologic malignancies predominated 60% of the study population, followed by solid tumors (14%) and overlapping solid/hematologic malignancies (14%). Additional details regarding malignancy history are presented in Supplementary Table 1.

**Table 1:** Baseline characteristics of the study cohort

Variables	N (%), Mean ± SD
Age, years	64.4 ± 9.0
Female sex	17 (60.7%)
**Race**	
1 Caucasian	19 (67.9%)
2 African American	8 (28.6%)
3 Other	1 (3.6%)
BMI, kg/m2	32.2 ± 8.1
Hypertension	22 (78.6%)
Hyperlipidemia	22 (78.6%)
Diabetes Mellitus Type II	10 (35.7%)
Tobacco history	11 (39.3%)
Coronary artery disease	15 (53.6%)
Chronic kidney disease	16 (57.1%)
Atrial Fibrillation/Flutter	11 (39.3%)
**Type of HF**	
1) HFrEF	10 (35.7%)
2) HFpEF	18 (64.3%)
**History of Pacemaker/ICD**	3 (10.7%), 2 (7.1%)
**History of Aortic Valve Replacement**	
1) TAVR	2 (7.1%)
2) SAVR	1 (3.6%)
**Types of Malignancy**	
• Hematological	17 (60.8%)
• Solid	4 (14.4%)
• Both Hematological/Solid	4 (14.4%)
• Others	3 (10.8%)
**Indications for CardioMEMS Placement**	
1) Amyloidosis	8 (28.6%)
2) HFpEF	5 (17.9%)
3) HFrEF	8 (28.6%)
4) Radiation-induced	6 (21.4%)
**Baseline Medications**	
1) ACEI/ARBs/ARNI	12 (42.8%)
2) Beta blockers	21 (75.0%)
3) Loop Diuretics	26 (92.9%)
4) Anticoagulation	12 (42.9%)
5) Antiplatelet therapy	13 (46.4%)

*SD* Standard Deviation, *HFrEF* Heart Failure with Reduced Ejection Fraction, *HFpEF* Heart Failure with Preserved Ejection Fraction, *TAVR* Transcatheter Aortic Valve Replacement, *SAVR* Surgical Aortic Valve Replacement, *ACEI* Angiotensin-converting enzyme inhibitors, *ARBs* Angiotensin receptor blockers, *ARNI* Angiotensin receptor/Neprilysin inhibitor

Table 2 shows patients’ baseline laboratory, echocardiographic, and hemodynamic parameters. Patients had a mean baseline creatinine of 1.8 (SD ± 1.1) mg/dl, and with a median NT-ProBNP level of 3065 pg/ml (IQR 617–11,131). The median baseline left ventricular ejection fraction (LVEF) was 54.4% (IQR 35.2–61.5%). Mean right atrial pressure (mRAP) was 13.2 (SD ± 6.4) mmHg, while mPAP was 35.1 (SD ± 10.0) mmHg and mean pulmonary capillary wedge pressure (PCWP) (mmHg) was 22.5 (SD ± 7.9) mmHg. Nine (33%) of patients had elevated transpulmonary gradient (TPG) (≥ 14 mm Hg).

**Table 2:** Baseline laboratory, echocardiographic, and hemodynamic parameters

Variable	Mean ± SD or Median (IQR)
**Laboratory values**	
Creatinine (mg/dL)	1.8 ± 1.1
Platelet count (k/uL)	222.0 ± 87.0
NT-proBNP (pg/ml)	3065 (617–11,131)
Troponin T (ng/L)	49 (34.5–84.5)
**Echo parameters**	
BSA (m^2^)	1.96 ± 0.28
LVEF (%)	54.4 (35.2–61.5)
LVEDV (ml)	129.8 (100.1–184.8)
LEVDI (ml/m^2^)	63.5 (52.0–87.2)
LVESV (ml)	53.5 (42.5–132.1)
LAV (ml)	76.3 ± 28.0
LAVI (ml/m^2^)	38.3 ± 13.0
**Hemodynamic parameters**	
mRAP, mmHg	13.2 ± 6.4
RVSP, mmHg	50.2 ± 12.4
PASP, mmHg	50.6 ± 11.5
PADP, mmHg	25.0 ± 8.3
mPAP, mmHg	35.1 ± 10.0
PCWP, mmHg	22.5 ± 7.9
PVR, WU	1.86 (0.99–4.52)
TPG, mmHg	12.3 ± 7.6
DPG, mmHg	1.4 ± 6.8
CO-TD, L/min	5.3 ± 2.2
CI, L/min/m^2^	2.8 ± 1.3

*IQR* Interquartile range, *BSA* body surface area, *LVEF* Left Ventricular ejection fraction, *LVEDV* left ventricular end diastolic volume, *LEVDVI* LVEDV index, *LVESV* left ventricular end systolic volume, *LAV* left atrial volume, *LAVI* LAV Index, *mRAP* mean right atrial pressure, *RSVP* right ventricular systolic pressure, *PASP* pulmonary artery systolic pressure, *PADP* pulmonary artery diastolic pressure, *mPAP* mean pulmonary artery pressure, *PCPW* pulmonary capillary wedge pressure, *PVR* pulmonary vascular resistance, *TPG* transpulmonary gradient, *WU* woods unit, *CO-TD* cardiac output by thermodilution, *CI* cardiac index

### Clinical outcomes and device safety

No patients experienced sensor failure (Table 3). One patient had a DSRC and required the deployment of a secondary device during the same procedure. During the study follow-up, seven patients experienced death, one of which was cardiac death.

**Table 3:** Procedural and patients’ outcomes

Variables	Number (%)
Device or System-Related Complications	1 (3.6%)
Pressure Sensory Failure	0
Death (not related to procedure)	7 (25%)
Cardiac Death	1 (3.6%)

### Heart failure hospitalization outcomes

**Fig. 1:**
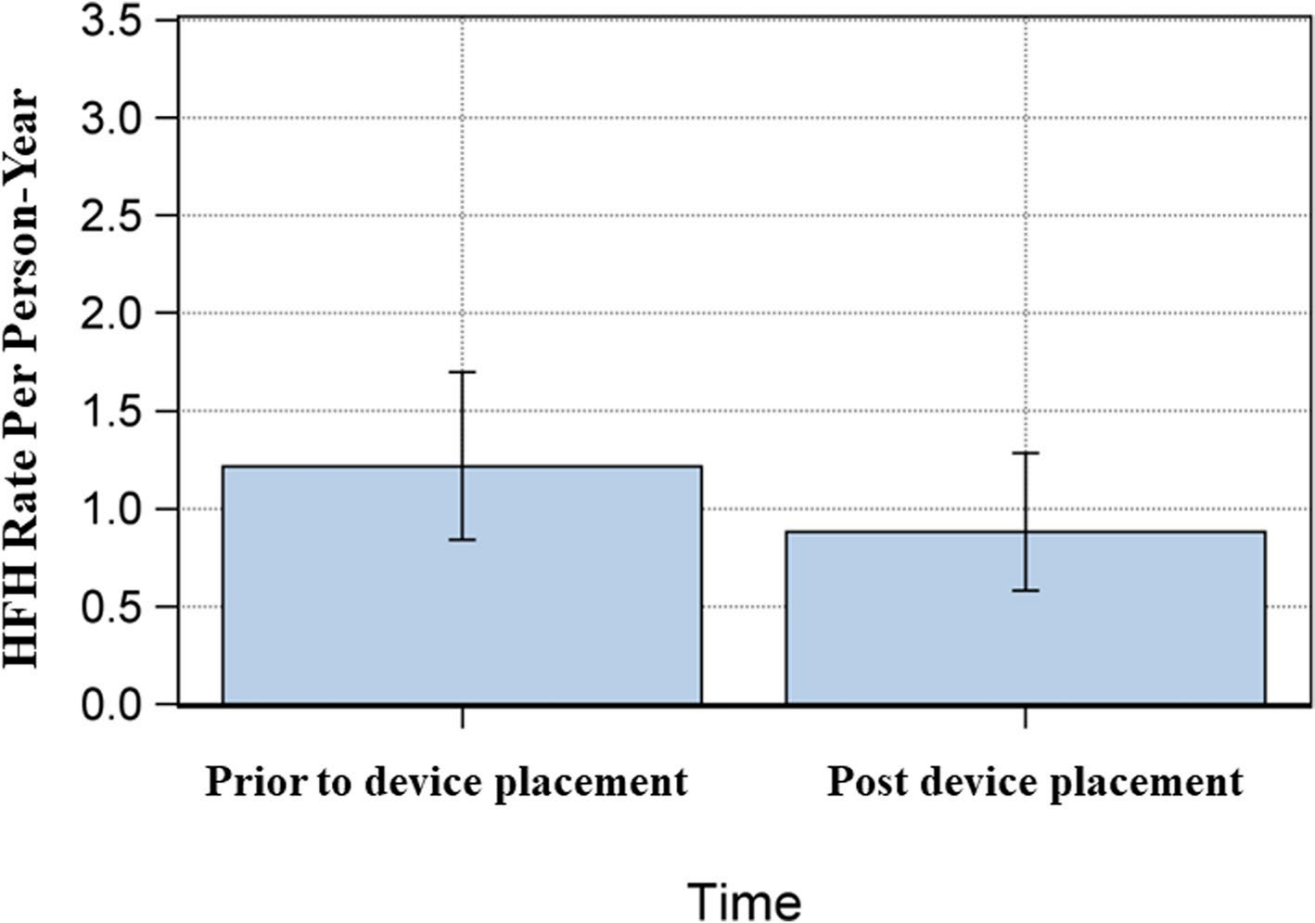
Number of HFHs before device implantation vs. HFHs after device implantation

Admission event rates per person-year, before and after the device was implanted are reported in Table 4. There was a reduction in HFH after device implantation (Fig. 1). This was observed across various heart failure phenotypes, except for radiation-induced heart disease. Patients with HFpEF experienced a higher number of hospitalizations compared to those with HFrEF, with a significantly greater median number of admissions (1 [range: 0–5] vs. 0 [range: 0–2], *p* = 0.036). However, the overall risk of hospitalization was not statistically different between the two groups (HR: 2.69, 95% CI: 0.85–8.54, *p* = 0.093).

**Table 4:** Number of HFH prior to device implantation VS. number of HFH admissions following device implantation

Variables	Total person years	Event Rate (CI: 95%)
**Timing**		
1. Prior to Device Implantation	27.98	1.22 (0.84–1.70)
2. Post Device Implantation	30.60	0.88 (0.58–1.28)
**Cardiac Indication**^**a**^		
1) Amyloidosis	10.78	0.74 (0.32–1.46)
2) HFpEF	10.14	0.79 (0.34–1.56)
3) HFrEF	3.61	0.83 (0.17–2.43)
4) Radiation-induced	5.90	1.19 (0.48–2.44)
Type of HF		
1) HFrEF	6.53	0.46 (0.09–1.34)
2) HFpEF	24.07	1.00 (0.64–1.48)

*CI* confidence interval, *HFrEF* Heart Failure with Reduced Ejection Fraction, *HFpEF* Heart Failure with Preserved Ejection Fraction

^a^One patient with suspected cardiac amyloidosis was not included

Cox regression models showed that higher number of prior HFH, elevated PAS, and PAD were associated with higher risk of HFH (Table 5). Additional Cox regression models demonstrated that prior HFH and abnormally elevated PAD were most strongly associated with increased risk of HFH after device placement (Supplementary Table 2). Mean cumulative number of HFH according to prior HFH, PAS, PAD, and mPAP are presented in Supplementary Fig. 1. Profile plots of PAD, and mPAP over time including those with and without HF admissions reveal that patients with HFH after device placement had higher device-measured PAD and mPAP than those without HFH after device placement (Supplementary Fig. 2).

**Table 5:** Univariable and multivariable recurrent event analysis for HFH after device implantation

Covariate	Level	Univariable		Multivariable	
		HR (95% CI)	*P*-value	HR (95% CI)	*P*-value
Age, years	1 Unit Change	1.00 (0.96–1.05)	0.89		
Sex	Female	2.13 (0.80–5.68)	0.13		
LVEF, mmHg	1 Unit Change	1.00 (0.99–1.03)	0.45		
mRAP, mmHg	1 Unit Change	1.07 (0.98–1.17)	0.12		
RVSP, mmHg	1 Unit Change	1.05 (1.02–1.08)	**0.003**		
Baseline PASP, mmHg	1 Unit Change	1.04 (1.01–1.07)	**0.004**		
Baseline PADP, mmHg	1 Unit Change	1.02 (0.98–1.07)	0.30		
Baseline mPAP, mmHg	1 Unit Change	1.11 (1.06–1.16)	** <.0001**		
Baseline PCWP, mmHg	1 Unit Change	1.00 (0.95–1.06)	0.88		
Baseline TPG, mmHg	1 Unit Change ≥ 14	1.08 (1.03–1.13) 3.80 (1.70–8.50)	**0.001** **0.0012**		
PVR, WU	1 Unit Change ≥ 2	1.19 (1.05–1.35) 3.11 (1.35–7.14)	**0.008** 0.008		
Prior HFH	1 Unit Change 0 1 2 ≥ 3	1.40 (1.22–1.60) 1.00 2.96 (1.01–8.70) 4.84 (1.43–16.43) 7.66 (2.82–10.70	** < 0.0001** **0.045** **0.045** ** < 0.0001**	1.24 (1.09–1.41)^a^	**0.0009**
Device Readings:					
1. PAS, mmHg	1 Unit Change > 35	1.06 (1.03–1.10) 4.23 (0.96–18.64)	** < 0.0001** 0.06	1.04 (1.01–1.07)^a^	**0.02**
2. PAD, mmHg	1 Unit Change > 18	1.13 (1.06–1.20) 5.74 (1.85–17.80)	**0.0001** **0.003**	1.07 (1.02–1.13)^a^	**0.006**
3. mPAP, mmHg	1 Unit Change > 20	1.11 (1.06–1.16) 6.21 (0.90–43.03)	** < 0.0001** 0.064		
Baseline NT-ProBNP	≥ 400	1.36 (0.62–2.98)	0.44		

^a^Model 1 including HFH, PAS and PAD

*HFH* Heart failure hospitalization, *LVEF* Left Ventricular ejection fraction, *mRAP* Mean right atrial pressure, *SRVP* Systolic right ventricular pressure, *PAS* Pulmonary artery systolic pressure, *PAD* Pulmonary artery diastolic pressure, *mPAP* Mean pulmonary artery pressure, *PCWP* pulmonary capillary wedge pressure, *PVR* Pulmonary vascular resistance, *TPG* Transpulmonary gradient, *WU* Woods unit

**Fig. 2:**
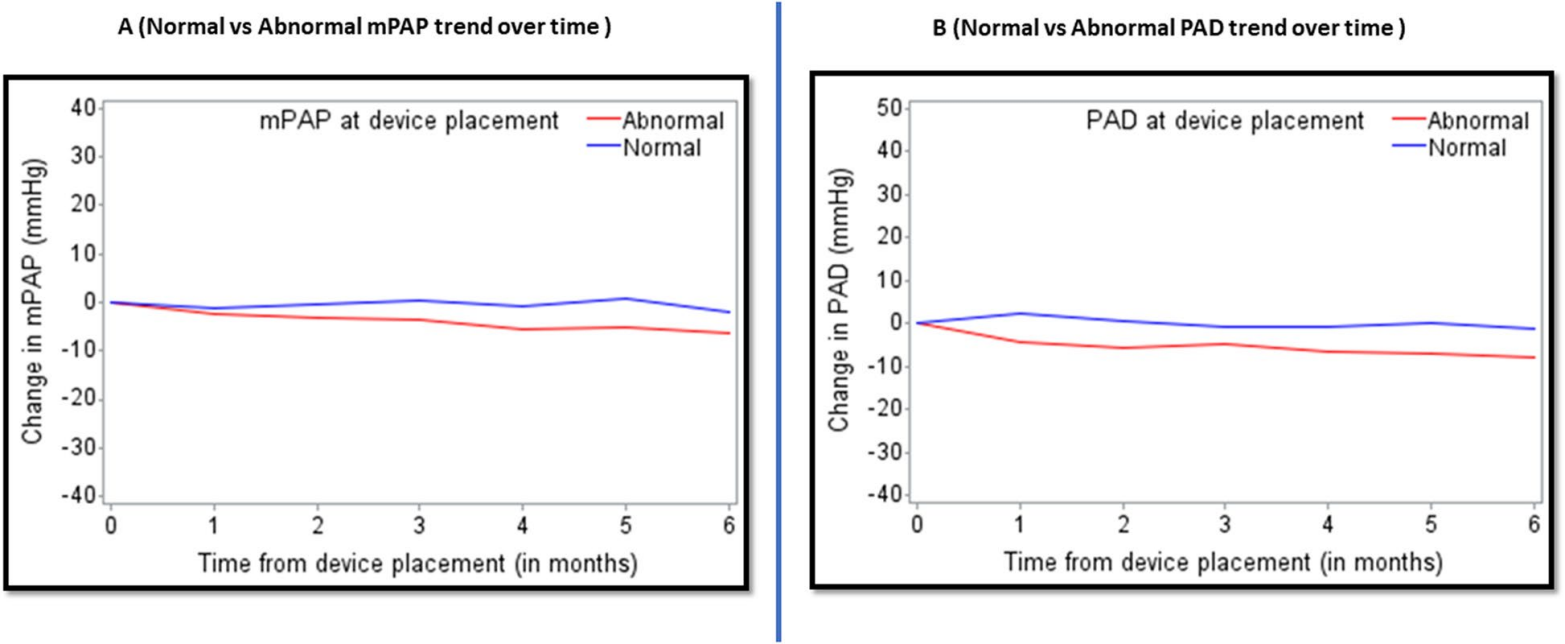
Hemodynamics changes over time. **A** mPAP trend over time. **B** PAD trend over time

Figure 2 depicts the mPAP trend over time from the time of device placement. Patients with abnormally elevated mPAP (> 20 mmHg) at the time of device placement experienced greater mPAP decline than did patients with normal baseline mPAP. Similarly, we observed greater reductions in PAD over time in patients with elevated baseline PAD (> 18 mmHg).

### Correlation between biomarker analysis and CardioMEMS hemodynamics

**Fig. 3:**
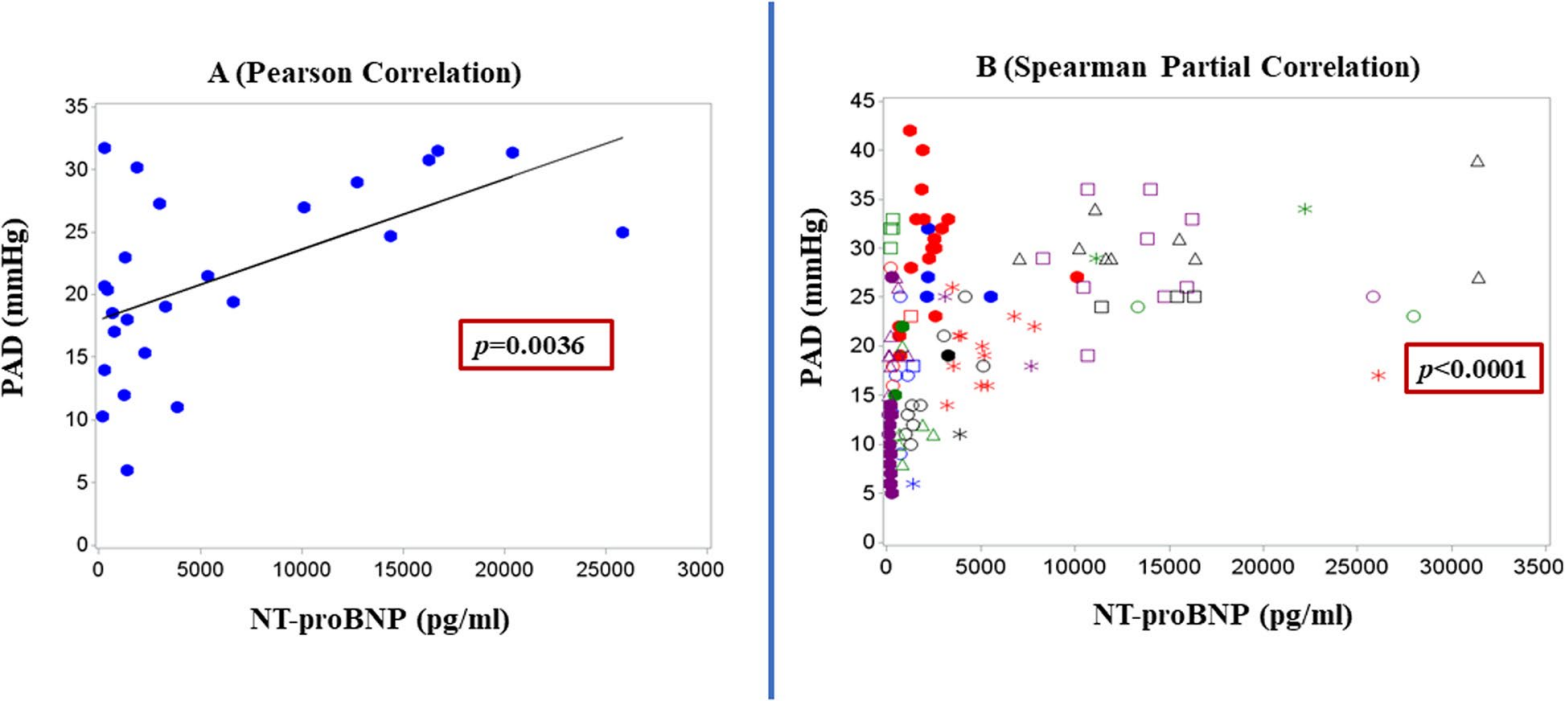
Correlation between NT-ProBNP and PAD. **A** Pearson correlation scatter plot of mean PADP and mean NT-ProBNP. **B** Spearman correlation scatter plot of PADP and NT-ProBNP

We assessed the relationship between CardioMEMS hemodynamics and serum natriuretic peptides. In Fig. 3, scatter plots of the mean PAD and mean NT-proBNP demonstrated a moderate positive correlation, with a Pearson correlation coefficient of 0.56 (*p* = 0.0036). Additionally, when adjusted for the effect of the subject, the Spearman partial correlation was 0.54 (*p* < 0.0001). Furthermore, mean mPAP and mean NT-proBNP, were moderately correlated with a Spearman correlation coefficient of 0.55 (*p* = 0.0045, Supplementary Fig. 3). The Spearman partial correlation, accounting for the subject effect in this case, was 0.60 (*p* < 0.0001).

To examine the impact of NT-proBNP and PAD on all-cause admissions and HFH following device implantation, we fitted two multivariable models for each outcome (any admission and HFH). Elevated NT-proBNP (> 400pg/mL) levels and PAD modeled as continuous variables, were significantly associated with increased risk for any hospital admission (HR: 3.05, *p* = 0.01; and HR: 1.08, *p* = 0.005, respectively (Supplementary Table 3.1, model 1). In model 2, elevated NT-proBNP values continue to demonstrate a significant association with increased risk of all-cause hospitalization (HR: 2.36, *p* = 0.0214), but elevated PAD (> 18 mmHg) as a categorical variable was not significantly associated with increased risk of admissions (HR:1.94, *p* = 0.24).

Similarly, PAD, modeled as a continuous variable, when adjusted for NT-proBNP was significantly associated with HFH (HR: 1.11, *p* = 0.0014, model 3). However, when both PAD and NT-proBNP were considered as a categorical variable, NT-proBNP was not significantly associated with HFH (HR: 1.55, *p* = 0.242) while PAD was marginally significant (HR: 3.2, *p* = 0.05).

To assess whether NT-proBNP retained prognostic value independent of renal function, we conducted a stratified analysis based on baseline creatinine levels (> 1.2 mg/dL vs. ≤ 1.2 mg/dL). In these stratified models, NT-proBNP was not significantly associated with HFH in either subgroup (Supplementary Table 3.2).

## Discussion

This study highlights the novel use of CardioMEMS for managing heart failure in a heterogenous cohort of cancer patients, highlighting the potential utility of PAPSD in mitigating risk of HFH. First, the study confirms the safety of CardioMEMS deployment in a complex patient population. Second, we observed a reduction in HFH following device implantation, indicating potential benefits in managing HF among these patients. Third, the data shows a consistent decline in PAD and mPAP during the follow-up period. Lastly, the study revealed a moderate correlation between NT-proBNP levels and PAD, suggesting that variations in NT-proBNP in cancer patients could be linked to changes in PAD.

### CardiMEMS system safety

Landmark randomized clinical trials have extensively evaluated the safety and efficacy of the CardioMEMS device in improving heart failure outcomes. The CHAMPION trial demonstrated a 98.6% rate of freedom from DSRC at 6 months, with no reported pressure sensor failures [10]. Subsequent studies, including GUIDE-HF, reported a 99% freedom from DSRC at 12 months, without any pressure sensor failures [11]. More recently, the MONITOR-HF trial showed a 97.7% freedom from DSRCs and a 98.8% rate of sensor reliability at 12 months [15]. Additionally, community-based data from an open-label observational study by Shavelle et al. revealed a 99.6% freedom from DSRC and a 99.9% freedom from pressure sensor failures [14]. In our study, freedom from DSRC was 96.4%: one patient required the deployment of a second device, which we attribute to our early experience with device implantation. We also did not witness any pressure sensor failures. These findings underscore the safety of the CardioMEMS device, even in our high-risk patient cohort.

### CardioMEMS Performance in high-risk populations

Despite the marked morbidity and mortality in oncology patients, cardiovascular care is often minimized with underutilization of guideline directed medical therapy for HF [26] and more invasive interventions like percutaneous coronary intervention [27]. Furthermore, concerns about cancer recurrence often preclude oncology patients with advanced heart failure from receiving heart transplants/left ventricular assist devices (LVADs) for CTRCD [28]. Thus, there is unmet need among cancer patients to optimize filling pressures to prevent heart failure hospitalizations. In our cohort, PAPSD monitoring was associated with declines in PAD and mPAP over time, along with a reduction in HFH.

It is important to note that our population included both HFpEF and HFrEF patients, similar to previous studies of CardioMEMS [10–12, 15]. However, our cohort also encompassed more heterogenous and complex pathologies including restrictive physiology from etiologies such as light chain amyloidosis and radiation induced heart disease. These subgroups have not been previously investigated in studies of invasive PAPSD technologies. A challenging dilemma in our population, was identifying the ideal, tolerable combination of guideline directed medical therapy to act on the data provided by CardioMEMS [26, 29, 30]. However, CardioMEMS has demonstrated utility in other physiologically complex populations, such as patients with LVADs. A recent study of such patients by Thohan et al. highlighted reductions in PAD at six months, improvements in the results of the six-minute walk test, and reduced HFH when PAD was maintained below 20 mmHg [31]. In our cohort, a third of the patients presented with an elevated TPG of ≥ 14mmHg, indicative of combined pre- and post-capillary pulmonary hypertension (Cpc-PH). The optimal management of these patients remains unclear, particularly regarding the reliability of PASDP in assessing left-sided filling pressures and mitigating heart failure exacerbations [32, 33]. Furthermore, our data revealed that patients with a PVR ≥ 2 WUs experienced similar rates of HFH compared to those with PVR < 2 WUs, suggesting a potential benefit of PASDP in patients with Cpc-PH. Future studies may help establish the benefits of PASDP in patients with Cpc-PH.

Furthermore, through multivariate regression modeling we identified prior HFH, and elevated device measured PAS and PAD (Table 5) as the strongest predictors of future HFH following CardioMEMS implantation. These findings highlight the association of higher device-measured pressures with future HFH, which underscores the importance of targeting lower PADP levels. Additionally, the reduction in post-implant HFH and greater reductions in device-measured PAD and mPAP over time among patients with elevated baseline PAD and mPAP highlights the potential benefit of tailored HF management in this patient population. Therefore, larger future prospective registries or RCTs are ultimately needed to confirm these benefits.

### NT-proBNP and cardioMEMS hemodynamics

There is no clear consensus on the use of cardiac biomarkers such as NT-proBNP in managing cardio-oncology patients; guidelines often rely on expert opinion [8, 34]. Oncology patients may exhibit elevated NT-proBNP levels due to factors such as disease burden [35]. Furthermore, even before cardiotoxic treatment, cancer patients can have elevated NT-proBNP (defined as NT-proBNP > 125 pg/mL) levels, which is significantly associated with all-cause mortality, highlighting the interplay between subclinical cardiac dysfunction with cancer progression [36]. GUIDE-HF was the only CardioMEMS randomized controlled trial that enrolled patients based on NT-proBNP values within 30 days before study consent, yet treatment effect heterogeneity based on NT-proBNP strata were not reported [11]. Additionally, our findings demonstrate that NT-proBNP correlates modestly with hemodynamic parameters (PAD and mPAP), supporting its value in managing patients with diverse cancer pathologies. This finding agrees with earlier studies conducted in populations of patients without cancer [37, 38]. However, it is crucial to consider these biomarkers in conjunction with the overall clinical presentation of the patients. This consideration is underscored by our observation that both NT-proBNP and PAD were associated with increased hospitalization, raising concerns that, despite competing risks for admission such as infection or cancer progression, many patients may be experiencing volume overload and pulmonary venous congestion. It is important to mention that the baseline creatinine level in our cohort was 1.8 mg/dl, which could have influenced the NT-proBNP values, as these are known to increase in patients with chronic kidney disease. Nonetheless, it still continues to hold a prognostic value [39, 40].

### Limitations

This study provides valuable insights, but we note several limitations. As a single-center retrospective analysis conducted at a tertiary cancer center, the findings might not be generalizable to broader patient populations. The small sample size, although oncologically diverse, limits the statistical power to detect smaller effects. Significant selection bias is present as our clinicians carefully identify patients for CardioMEMS implantation. Furthermore, as some patients might return to their local healthcare providers after completing cancer treatment, hospital event rates may be underestimated if not captured in the electronic health record. Additionally, the relatively short median follow-up period of 8.8 months may not adequately reflect the long-term outcomes of device implantation in managing heart failure. We also acknowledge the potential impact of competing risks, particularly cancer-related mortality, on heart failure hospitalization outcomes. Our primary objective was to assess hospitalization burden and temporal trends during active follow-up, and therefore a competing risks model was not applied.

### Future directions

The future of cancer care lies in personalized precision medicine. Although our study involves a small and diverse sample, this is the first published report of invasive PAPSD use in a real-world cardio-oncology patient cohort. Given the challenges of conducting randomized trials in such a heterogenous and high-risk group of patients, future collaborative efforts, including the development of prospective registries or pragmatic trials is necessary to identify how to leverage these technologies to reduce morbidity. Additionally, longer follow-up periods will be crucial to identify the long-term efficacy of such tailored care and its overall impact on patient outcomes. Furthermore, integrating device data with cancer treatment plans, exploring new biomarkers for better risk assessment, advancing device technology, and assessing preventive applications before the onset of CTRCD are all potential topics of future inquiry. Furthermore, this unique interdisciplinary approach is well-placed to incorporate patient experiences and patient-focused outcomes as new technologies align with patient-centered healthcare models.

## Conclusions

Our study suggests that the CardioMEMS holds promise for managing heart failure in patients undergoing cancer treatment, a group traditionally underrepresented in cardiovascular research. Importantly, while our sample primarily was composed of Caucasian individuals, these findings suggest that the impact of heart failure management might be even more pronounced in African American and Hispanic populations. Additionally, our results confirmed the safety of the device in a higher-risk patient population, with a high rate of freedom from device/system-related complications. Moreover, we observed a reduction in HFHs and consistent improvements in key hemodynamic parameters such as PAD and mPAP. Ultimately, this research moves us closer to a more personalized and proactive approach in the management of cardio-oncology patients, promoting better patient outcomes through advanced technologies and integrated care strategies. These results encourage further investigation into the use of invasive PAPSD in patients with cancer, ideally through larger, multi-center prospective studies that can provide more robust data to confirm these preliminary findings and shape future clinical practice.

## Supplementary Information

Supplementary Material 1

## Data Availability

No datasets were generated or analysed during the current study.

## Abbreviations

HF: Heart failure
HFH: Heart failure hospitalization
PAS: Pulmonary artery systolic pressure
PAD: Pulmonary artery diastolic pressure
PAPSD: Pulmonary artery pressure sensing devices
HFrEF: Heart failure with reduced ejection fraction
HFpEF: Heart failure with preserved ejection fraction
CTRCD: Cancer therapy related cardiac dysfunction
