# Supplementary material for: CardioMEMS guided heart failure management in cardio-oncology patients: a tertiary care cancer center experience
Source: Cardiooncology. 2025 Jul 18;11:67. doi: 10.1186/s40959-025-00355-0 (PMC12273344; doi:10.1186/s40959-025-00355-0)
Supplement: Supplementary file 1 — Supplementary Material 1 [file 40959_2025_355_MOESM1_ESM.docx]

**Supplementary Table 1. Distribution of Malignancy Types Among Study Cohort**

| **Patient** | **Malignancy Type** | **Radiation Exposure** | **Anthracycline Exposure** |
| --- | --- | --- | --- |
| **1** | Multiple myeloma | **-** | **-** |
| **2** | Multiple myeloma | **-** | **-** |
| **3** | Multiple myeloma | **-** | **-** |
| **4** | Multiple myeloma | **-** | **-** |
| **5** | Multiple myeloma | **-** | **-** |
| **6** | Multiple myeloma | **-** | **-** |
| **7** | Light chain (AL) amyloidosis | **-** | **-** |
| **8** | Light chain (AL) amyloidosis | **-** | **-** |
| **9** | Light chain (AL) amyloidosis | **-** | **-** |
| **10** | Suspected amyloidosis | **-** | **-** |
| **11** | Large cell lymphoma | **-** | **+** |
| **12** | Hodgkin lymphoma | **+** | **+** |
| **13** | Hodgkin lymphoma | **+** | **-** |
| **14** | Breast and prior lymphoma | **+** | **-** |
| **15** | Lymphoma and breast cancers | **+** | **-** |
| **16** | Follicular lymphoma | **-** | **+** |
| **17** | Hodgkin lymphoma | **+** | **+** |
| **18** | Lymphoma, prostate cancer | **+** | **+** |
| **19** | Acute myelogenous leukemia | **-** | **-** |
| **20** | Chronic myeloid Leukemia /renal cell carcinoma | **-** | **-** |
| **21** | Chronic myeloid Leukemia | **-** | **-** |
| **22** | Chronic myeloid Leukemia | **-** | **-** |
| **23** | Lung cancer | **+** | **-** |
| **24** | Duodenal neuroendocrine tumor | **-** | **-** |
| **25** | Breast and bladder cancers | **-** | **-** |
| **26** | Bladder cancer | **-** | **-** |
| **27** | Suspected lung cancer with pulmonary nodules | **-** | **-** |
| **28** | Suspected gastrointestinal malignancy | **-** | **-** |

**Supplementary Table 2. Additional Multivariable Modeling for Recurrent Event Analysis, focusing on HFH admissions following device Implantation.**

| **Multivariable Model** | **Covariate** | **Level** | **HR (95% CI)** | **P-value** |
| --- | --- | --- | --- | --- |
| **Model 2** | Prior HFH | 1 | 2.93 (0.70-12.26) | 0.14 |
|  |  | 2 | 2.50 (0.69-9.08) | 0.17 |
|  |  | ≥3 | 5.18 (1.82-14.78) | **0.002** |
|  | PAS | 1 Unit Change | 1.04 (1.01-1.07) | **0.009** |
|  | PAD | 1 Unit Change | 1.07 (1.03-1.12) | **0.002** |
| **Model 3** | Prior HFH | 1 Unit Change | 1.28 (1.11-1.48) | **0.001** |
|  | PAD | ≤18 | 1.000 |  |
|  |  | >18 | 4.52 (1.41-14.56) | **0.011** |
| **Model 4** | Prior HFH | 1 | 2.28 (0.53-9.82) | 0.27 |
|  |  | 2 | 3.15 (0.91-10.89) | 0.07 |
|  |  | ≥3 | 5.07 (1.93-13.37) | **0.001** |
|  | PAD | >18 | 4.06 (1.32-12.53) | **0.015** |

HFH: heart failure hospitalization, HR: Hazards ratio, PAS: pulmonary artery systolic pressure, PADP: pulmonary artery diastolic pressure.

**Supplementary Table 3.1. Predictive Value of NT-ProBNP and PAD for Post-Device Placement Hospital Admissions.**

| **Multivariable Model** | **Covariate** | **Level** | **HR (95% CI)** | **P-value** |
| --- | --- | --- | --- | --- |
| **Model 1#** | NT-ProBNP | ≥400 pg/mL | 3.05 (1.27-7.31) | **0.013** |
|  | PAD | 1 unit change | 1.08 (1.02-1.14) | **0.005** |
|  |  |  |  |  |
| **Model 2#** | NT-ProBNP | ≥400 pg/mL | 2.36 (1.14-4.89) | **0.021** |
|  | PAD | >18 mmHg | 1.94 (0.64-5.96) | 0.24 |
|  |  |  |  |  |
| **Model 3** | NT-ProBNP | ≥400 pg/mL | 1.89 (0.90-3.97) | 0.095 |
|  | PAD | 1 unit change | 1.11 (1.04-1.18) | **0.001** |
| **Model 4** | NT-ProBNP | ≥400 pg/mL | 1.55 (0.75-3.20) | 0.242 |
|  | PAD | >18 mmHg | 3.19 (1.00-10.17) | 0.050 |

* In the biomarker data, there were only 4 cases with proBNP<125. Therefore, we only used 400 as a cutoff value. PADP: pulmonary artery diastolic pressure. #Models 1 and 2 are for predicting any hospital admission. Models 3 &4 are for predicting any HFH.

**Supplementary Table 3.2. Predictive Value of NT-ProBNP and PAD for Post-Device Placement Hospital Admissions stratified by renal dysfunction.**

| **Multivariable Model** | **Covariate** | **Level** | **HR (95% CI)** | **P-value** |
| --- | --- | --- | --- | --- |
| **Model 1#** | NT-ProBNP | ≥400 pg/mL | 2.09 (0.91-4.80) | 0.08 |
|  | PAD | 1 unit change | 1.08 (1.02-1.16) | **0.014** |
|  |  |  |  |  |
| **Model 2#** | NT-ProBNP | ≥400 pg/mL | 1.86 (0.64-5.41) | 0.26 |
|  | PAD | >18 mmHg | 2.80 (0.92-8,51) | 0.07 |
|  |  |  |  |  |
| **Model 3** | NT-ProBNP | ≥400 pg/mL | 1.43 (0.78-2.62) | 0.25 |
|  | PAD | 1 unit change | 1.07 (1.01-1.14) | **0.02** |
| **Model 4** | NT-ProBNP | ≥400 pg/mL | 1.09 (0.54-2.24) | 0.80 |
|  | PAD | >18 mmHg | 2.91 (1.02-8.31) | 0.046 |

**Supplementary Figure 1. Mean cumulative number of HFH after device placement Stratified by prior (*A*) HFH, (*B*) PAD, (*C*) mPAP and (*D*) PAS.**

**
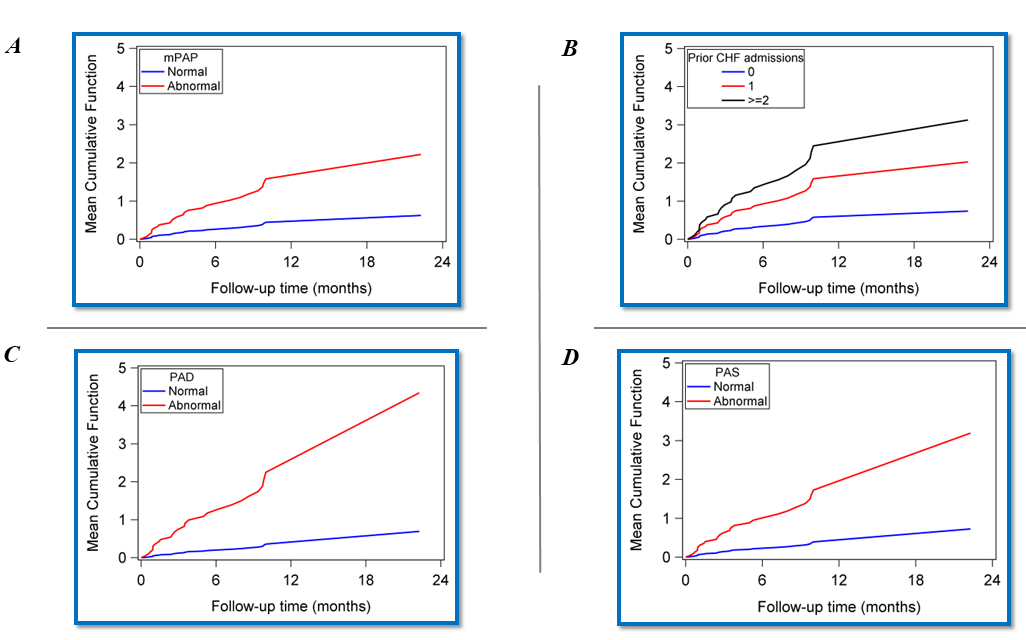
**

**Supplementary Figure 2. Profile plots over time of PAD (*A*) without and (*B*) with HFH after device placement and of mPAP (*C*) without and (*D*) with HFH after device placement


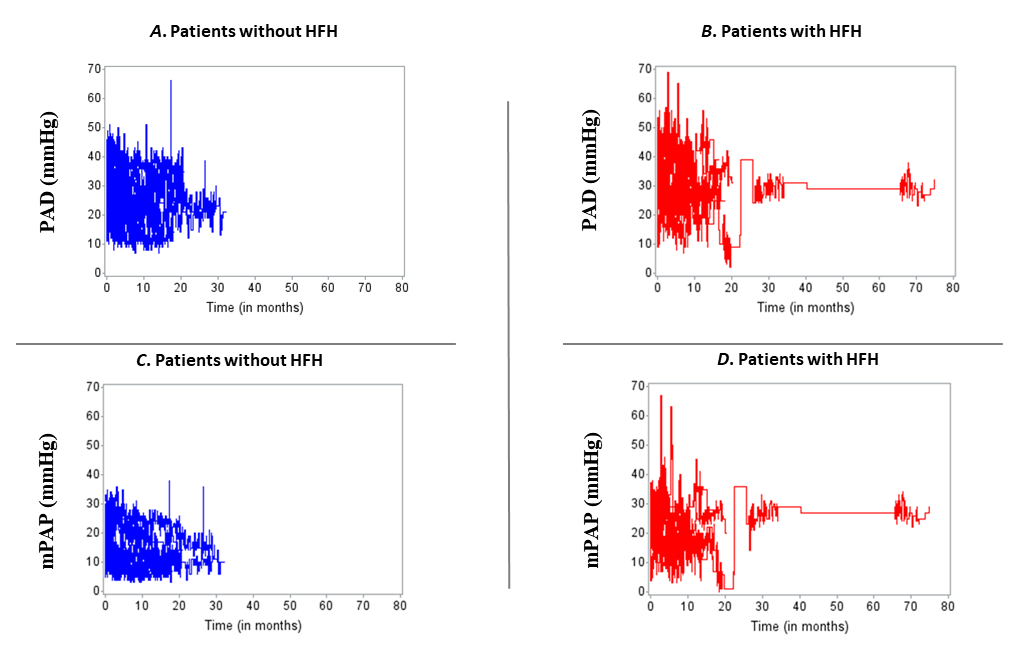
**

**Supplementary Figure 3. Spearman correlation between NT-ProBNP and mPAP. (*A*) Scatter plot of mean mPAP and mean NT-ProBNP. (*B*) Scatter plot of mPAP and NT-ProBNP.**

**
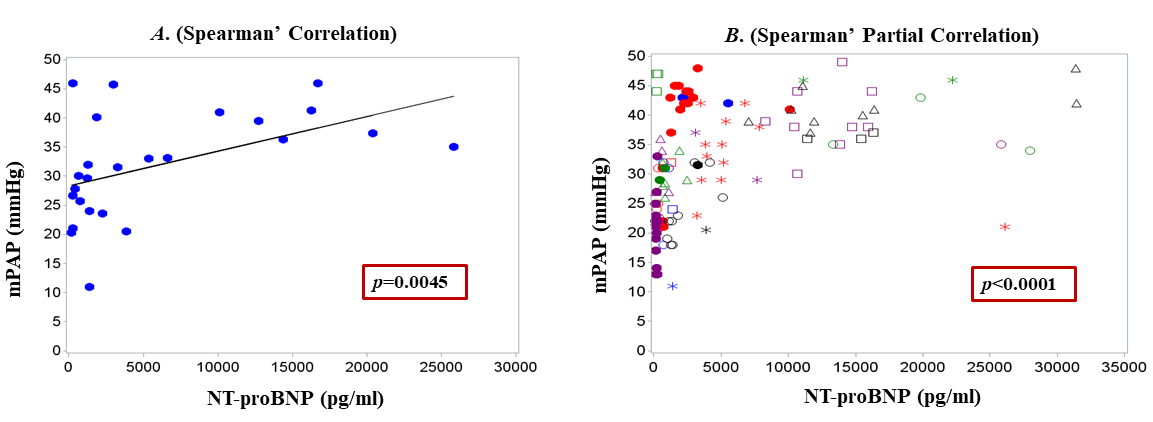
**

1.Agarwal MA, Fonarow GC, Ziaeian B. National Trends in Heart Failure Hospitalizations and Readmissions From 2010 to 2017. JAMA Cardiol 2021;6:952-956.

2.Matetic A, Mohamed M, Miller RJH et al. Impact of cancer diagnosis on causes and outcomes of 5.9 million US patients with cardiovascular admissions. Int J Cardiol 2021;341:76-83.

3.Strongman H, Gadd S, Matthews A et al. Medium and long-term risks of specific cardiovascular diseases in survivors of 20 adult cancers: a population-based cohort study using multiple linked UK electronic health records databases. Lancet 2019;394:1041-1054.

4.The global burden of cancer attributable to risk factors, 2010-19: a systematic analysis for the Global Burden of Disease Study 2019. Lancet 2022;400:563-591.

5.Koene RJ, Prizment AE, Blaes A, Konety SH. Shared Risk Factors in Cardiovascular Disease and Cancer. Circulation 2016;133:1104-14.

6.de Wit S, Glen C, de Boer RA, Lang NN. Mechanisms shared between cancer, heart failure, and targeted anti-cancer therapies. Cardiovasc Res 2023;118:3451-3466.

7.Kobo O, Raisi-Estabragh Z, Gevaert S et al. Impact of cancer diagnosis on distribution and trends of cardiovascular hospitalizations in the USA between 2004 and 2017. Eur Heart J Qual Care Clin Outcomes 2022;8:787-797.

8.Lyon AR, López-Fernández T, Couch LS et al. 2022 ESC Guidelines on cardio-oncology developed in collaboration with the European Hematology Association (EHA), the European Society for Therapeutic Radiology and Oncology (ESTRO) and the International Cardio-Oncology Society (IC-OS). Eur Heart J 2022;43:4229-4361.

9.Hospital Readmissions Reduction Program (HRRP).

10.Abraham WT, Adamson PB, Bourge RC et al. Wireless pulmonary artery haemodynamic monitoring in chronic heart failure: a randomised controlled trial. Lancet 2011;377:658-66.

11.Lindenfeld J, Zile MR, Desai AS et al. Haemodynamic-guided management of heart failure (GUIDE-HF): a randomised controlled trial. Lancet 2021;398:991-1001.

12.Adamson PB, Abraham WT, Bourge RC et al. Wireless pulmonary artery pressure monitoring guides management to reduce decompensation in heart failure with preserved ejection fraction. Circ Heart Fail 2014;7:935-44.

13.Adamson PB, Abraham WT, Stevenson LW et al. Pulmonary Artery Pressure-Guided Heart Failure Management Reduces 30-Day Readmissions. Circ Heart Fail 2016;9.

14.Shavelle DM, Desai AS, Abraham WT et al. Lower Rates of Heart Failure and All-Cause Hospitalizations During Pulmonary Artery Pressure-Guided Therapy for Ambulatory Heart Failure: One-Year Outcomes From the CardioMEMS Post-Approval Study. Circ Heart Fail 2020;13:e006863.

15.Brugts JJ, Radhoe SP, Clephas PRD et al. Remote haemodynamic monitoring of pulmonary artery pressures in patients with chronic heart failure (MONITOR-HF): a randomised clinical trial. Lancet 2023;401:2113-2123.

16.Lindenfeld J, Costanzo MR, Zile MR et al. Implantable Hemodynamic Monitors Improve Survival in Patients With Heart Failure and Reduced Ejection Fraction. J Am Coll Cardiol 2024;83:682-694.

17.Mokri H, Clephas PRD, de Boer RA, van Baal P, Brugts JJ, Rutten-van Mölken M. Cost-effectiveness of remote haemodynamic monitoring by an implantable pulmonary artery pressure monitoring sensor (CardioMEMS-HF system) in chronic heart failure in the Netherlands. Eur J Heart Fail 2024.

18.Cowie MR, Thokala P, Ihara Z, Adamson PB, Angermann C. Real-time pulmonary artery pressure monitoring in heart failure patients: an updated cost-effectiveness analysis. ESC Heart Fail 2023;10:3046-3054.

19.Adamson PB, Abraham WT, Aaron M et al. CHAMPION trial rationale and design: the long-term safety and clinical efficacy of a wireless pulmonary artery pressure monitoring system. J Card Fail 2011;17:3-10.

20.Brugts JJ, Veenis JF, Radhoe SP et al. A randomised comparison of the effect of haemodynamic monitoring with CardioMEMS in addition to standard care on quality of life and hospitalisations in patients with chronic heart failure : Design and rationale of the MONITOR HF multicentre randomised clinical trial. Neth Heart J 2020;28:16-26.

21.Lindenfeld J, Abraham WT, Maisel A et al. Hemodynamic-GUIDEd management of Heart Failure (GUIDE-HF). Am Heart J 2019;214:18-27.

22.Kanelidis AJ, Raikhelkar J, Kim G et al. CardioMEMS-Guided CAR T Cell Therapy for Lymphoma in a Patient With Anthracycline-Induced Cardiomyopathy. JACC CardioOncol 2020;2:515-518.

23.Khan MS, Khouri MG, Gomez L, Fudim M. Pressures do not equal volumes: implications for heart failure management in patients with CardioMEMS. ESC Heart Fail 2023;10:716-720.

24.PRENTICE RL, WILLIAMS BJ, PETERSON AV. On the regression analysis of multivariate failure time data. Biometrika 1981;68:373-379.

25.Welsh P, Campbell RT, Mooney L et al. Reference Ranges for NT-proBNP (N-Terminal Pro-B-Type Natriuretic Peptide) and Risk Factors for Higher NT-proBNP Concentrations in a Large General Population Cohort. Circ Heart Fail 2022;15:e009427.

26.Coles B, Welch CA, Motiwale RS et al. Acute heart failure presentation, management, and outcomes in cancer patients: a national longitudinal study. Eur Heart J Acute Cardiovasc Care 2023;12:315-327.

27.Bharadwaj A, Potts J, Mohamed MO et al. Acute myocardial infarction treatments and outcomes in 6.5 million patients with a current or historical diagnosis of cancer in the USA. Eur Heart J 2020;41:2183-2193.

28.Tie H, Zhu J, Akin S et al. Characteristics and Outcome of Patients With a History of Cancer Undergoing Durable Left Ventricular Assist Device Implantation. Circ Heart Fail 2023;16:e009772.

29.Irizarry-Caro JA, Song J, Miller C et al. Evaluation of Midodrine Utilization in Patients with Cancer and Heart Failure. Cardiovasc Drugs Ther 2024.

30.Wechalekar AD, Fontana M, Quarta CC, Liedtke M. AL Amyloidosis for Cardiologists: Awareness, Diagnosis, and Future Prospects: JACC: CardioOncology State-of-the-Art Review. JACC CardioOncol 2022;4:427-441.

31.Thohan V, Abraham J, Burdorf A et al. Use of a Pulmonary Artery Pressure Sensor to Manage Patients With Left Ventricular Assist Devices. Circ Heart Fail 2023;16:e009960.

32.Frantz RP. Hemodynamic monitoring in pulmonary arterial hypertension. Expert Rev Respir Med 2011;5:173-8.

33.Frantz RP, Benza RL, Kjellström B et al. Continuous hemodynamic monitoring in patients with pulmonary arterial hypertension. J Heart Lung Transplant 2008;27:780-8.

34.Pudil R, Mueller C, Čelutkienė J et al. Role of serum biomarkers in cancer patients receiving cardiotoxic cancer therapies: a position statement from the Cardio-Oncology Study Group of the Heart Failure Association and the Cardio-Oncology Council of the European Society of Cardiology. Eur J Heart Fail 2020;22:1966-1983.

35.Kamai T, Tokura Y, Uematsu T et al. Elevated serum levels of cardiovascular biomarkers are associated with progression of renal cancer. Open Heart 2018;5:e000666.

36.Pavo N, Raderer M, Hülsmann M et al. Cardiovascular biomarkers in patients with cancer and their association with all-cause mortality. Heart 2015;101:1874-80.

37.Knebel F, Schimke I, Pliet K et al. NT-ProBNP in acute heart failure: correlation with invasively measured hemodynamic parameters during recompensation. J Card Fail 2005;11:S38-41.

38.Souza R, Jardim C, Julio Cesar Fernandes C, Silveira Lapa M, Rabelo R, Humbert M. NT-proBNP as a tool to stratify disease severity in pulmonary arterial hypertension. Respir Med 2007;101:69-75.

39.Ozkan B, Grams ME, Coresh J et al. Associations of N-terminal pro-B-type natriuretic peptide, estimated glomerular filtration rate, and mortality in US adults. Am Heart J 2023;264:49-58.

40.Takase H, Dohi Y. Kidney function crucially affects B-type natriuretic peptide (BNP), N-terminal proBNP and their relationship. Eur J Clin Invest 2014;44:303-8.
